# FODMAP Content Like-by-like Comparison in Spanish Gluten-free and Gluten-containing Cereal-based Products

**DOI:** 10.1007/s11130-024-01177-8

**Published:** 2024-04-20

**Authors:** Silvia Matias, Gesala Perez-Junkera, Olaia Martínez, Jonatan Miranda, Idoia Larretxi, Lidia Peña, María Ángeles Bustamante, Itziar Churruca, Edurne Simón

**Affiliations:** 1 GLUTEN3S Research Group, Department of Pharmacy and Food Science, University of the Basque Country (UPV/EHU), Vitoria-Gasteiz, 01006 Spain; 2 Gluten Analysis Laboratory, Department of Pharmacy and Food Science, Faculty of Pharmacy, University of the Basque Country (UPV/EHU), University of the Basque Country (UPV/EHU), Paseo de la Universidad, 7, Vitoria- Gasteiz, 01006 Spain; 3 Nutrition and Food Safety Research Group, Bioaraba Health Research Institute, Vitoria- Gasteiz, 01006 Spain; 4 Ayuntamiento de Vitoria-Gasteiz, Centro Integral de Atención a Mayores San Prudencio, Vitoria-Gasteiz, 01006 Spain

**Keywords:** FODMAP, Gluten-free, Cereal, Coeliac, Individually wrapped

## Abstract

Gluten-free foods (GF) availability on supermarket shelves is growing and it is expected to continue expanding in the years ahead. These foods have been linked to a lower content of fermentable oligosaccharides, disaccharides, monosaccharides and polyols (FODMAPs), molecules that trigger gastrointestinal symptoms in sensitive persons. In this study, the FODMAP content of 25 cereal-based GF foods in Spain (breakfast cereals, pasta, bread, biscuits, bakery products, and dough and puff pastry) and 25 gluten-containing equivalents (GC) available in the same supermarket were analysed and compared. Lactose, fructose, glucose, sorbitol, mannitol, raffinose, stachyose and fructans were quantified. In a like-by-like analysis, GF foods were found to generally contain fewer FODMAPs than their GC counterparts. The ingredients used in the manufacture of GF cereal-based foods may contribute to this fact. When the individually wrapped size was considered, the proportion of samples classified as high-FODMAPs in GC and GF foods showed a trend towards fewer samples in the GF. However, not all the GF samples were low-FODMAP. Altogether, our findings provide essential information for FODMAP content databases of GF products in Spain.

**Supplementary Information:**

The online version contains supplementary material available at 10.1007/s11130-024-01177-8.

## Introduction

One of the most prevalent health concerns in current Western societies is that of adverse reactions to food intake. One in five people suffer from functional, non-specific, non-allergic gastrointestinal symptoms associated with intolerance/malabsorption of carbohydrates (lactose and fructose) or proteins (gluten), among other dietary components [1]. In terms of gluten exposure, approximately 5% of the population has problems related to this protein [2], most notably people diagnosed with coeliac disease (CD) or non-coeliac gluten sensitivity (NCGS).

This fact, coupled with the fad for GF foods [3], has led to the exponential growth in the gluten-free market, with a projected compound annual growth rate of 9.8% from 2022 to 2030 [4]. However, it is increasingly common to consider that other ingredients than gluten may also aggravate or be responsible for unpleasant gastrointestinal symptoms in sensitive people with CD or NCGS. This is the case for fermentable oligosaccharides, disaccharides, monosaccharides and polyols, FODMAPs [5]. The term ‘FODMAP’ refers to a group of five subgroups of poorly absorbed and rapidly fermented carbohydrates that are believed to cause gastrointestinal symptoms [6]. These subgroups include fructans, galacto-oligosaccharides, lactose, excess fructose, and polyols. The significance of these molecules from a dietetic perspective has increased, as low-FODMAP diet has been shown to be effective in treating the symptoms of irritable bowel syndrome (IBS) [7, 8].The mechanisms by which FODMAPs exert their effects range from osmotic and colonic fermentation observed in healthy individuals to those recently described in patients with IBS, which links these molecules to modulation of visceral hypersensitivity, neural or hormonal involvement, increase in intestinal permeability, induction of microbiota changes and production of short chain fatty acids (SCFAs), as well as metabolomics and alterations in motility [9]. Although they have common characteristics, the subgroups have differences in absorption and fermentation, and thus diverse potential mechanisms of action has been attributed to the low-FODMAP diet [6].

FODMAPs can be found in different concentrations within specific fruits, vegetables, legumes, dairy products, cereals, artificial sweeteners, and nuts [6]. However, FODMAP content in foods has not been widely studied in many countries, so increasing knowledge is essential for the design of low FODMAP diets, but also to enable consumers to choose more suitable products. Furthermore, for an accurate evaluation, country-specific databases should be made for health professionals [7, 10]. In fact, the European scientific community has started demanding specific labelling of foods low in FODMAPs, as occurs in countries such as Australia [11]. This requires specific analyses of FODMAP composition of foods available in the local market, as it has been performed for general-purpose foods in countries such as the United Kingdom and Sweden [12, 13]. In Spain, only one approach has been conducted in this regard. A study found and quantified the presence of gluten and fructans in industrial and artisanal breads [14].

Sainsbury et al. (15) reported in a meta-analysis that 38% of treated CD patients showed symptomatology compatible with IBS and that, although better adherence to the GF diet could reduce this percentage, they highlighted the fact that some other patients continue suffering from IBS-like symptoms despite extract adherence to the diet [15, 16]. In NGSC, fructans rather than gluten may promote gastrointestinal symptomatology [17]. This evidence supports the interest in finding out FODMAP content of GF products.

Several articles have suggested in a non-systematic way that GF foodstuffs may contain less FODMAP than their equivalents [10, 18]. The confirmation of this general assumption with statistics could be of great help for people with intestinal function problems in the choice of supermarket foods. It is remarkable that, apart from people suffering from CD, there is significant adherence to the GF diet in Western societies, reaching up to 50% for specific population groups [19].

In order to confirm this hypothesis, the present work aims to systematically compare the FODMAP composition (lactose, fructose, glucose, sorbitol, mannitol, raffinose, stachyose and total fructans) of the most commonly consumed GF labelled cereal-based products in Spain with their equivalent GC options. In addition, the categorisation of high-FODMAP foods has traditionally been based on the weight of standardised servings per country [10]. The present research wants to broaden the dietary view by considering also other units of consumption such as individually wrapped size or maximum consumption.

## Materials and methods

The Materials and Methods section is presented as supplementary 1.

## Results and Discussion

Although the market for gluten-free foods is growing, there is currently a lack of data available on the FODMAP content of such foods in Spain. Additionally, no systematic comparison has been made in Spain between cereal-based GF foods and their gluten-containing counterparts. This study aims to fill this gap. Table 1 and supplementary 1 and 2 present the results of the FODMAP analyses performed. The only study conducted in Spain on gluten-free breads found that fructan levels for wheat-based breads sold in supermarkets in Spain ranged from 0.19 to 0.59 expressed as grams per 100 g of dry matter (which would be equivalent to 0.14–0.39 g of fructans per 100 g of edible portion of food (EPF) considering its moisture content) [14]. These values have been fully confirmed in the present study, since for the GC breads the fructan content expressed per 100 g of EPF was in the range of 0.14–0.38 g. The new feature of present research is that a comparison was made with an equivalent GF bread from the same store. The fructan content of breads in the GF group was 0.09 ± 0.21 g /100 g EPF (in addition to the abovementioned breads, toasted bread was included, range of 0.00–0.56 g), significantly lower than that of GC (*p* < 0.01). The same tendency was found for another oligosaccharide such as raffinose (0.14 ± 0.08 g /100 g for GC vs. 0.03 ± 0.04 g /100 g for GF), for the sorbitol polyol (0.01 ± 0.00 g /100 g for GC vs. 0.00 ± 0.01 g /100 g for GF) and for excess fructose (0.29 ± 0.23 g /100 g for GC vs. 0.03 ± 0.05 g /100 g for GF), calculated as the subtraction of fructose minus glucose, and considered as a FODMAP due to its potential risk of malabsorption [20]. The average amounts of lactose found were very low (4 mg/100 g for GC vs. 1 mg/100 g for GF). Similarly, the levels of mannitol or stachyose were very low and no significant differences were observed.

**Table 1:** Analytical excess fructose, sorbitol, total fructan, raffinose content in gluten-free foodstuffs divided by groups, compared to gluten-containing products

Food group	Excess Fructose			Sorbitol			Total fructan			Raffinose		
	GC	GF	p	GC	GF	p	GC	GF	p	GC	GF	p
Breakfast cereals	0.09 ± 0.15	0.00 ± 0.00	0.14	0.45 ± 0.56	1.26 ± 1.92	0.52	0.36 ± 0.17	0.29 ± 0.23	0.52	0.11 ± 0.04	0.11 ± 0.08	0.42
Pasta	0.00 ± 0.00	0.00 ± 0.00	1.00	0.00 ± 0.00	0.02 ± 0.01	< 0.05	0.70 ± 0.06	0.06 ± 0.07	< 0.05	0.08 ± 0.05	0.04 ± 0.01	0.25
Bread	0.29 ± 0.23	0.03 ± 0.05	< 0.01	0.01 ± 0.00	0.00 ± 0.01	< 0.05	0.31 ± 0.10	0.09 ± 0.21	< 0.01	0.14 ± 0.08	0.03 ± 0.04	< 0.001
Biscuits	0.00 ± 0.00	0.00 ± 0.00	1.00	0.00 ± 0.00	0.01 ± 0.01	0.14	1.04 ± 0.09	1.82 ± 2.52	0.33	0.20 ± 0.05	0.15 ± 0.14	0.42
Bakery	0.17 ± 0.30	0.09 ± 0.21	0.36	0.52 ± 0.64	0.15 ± 0.25	< 0.05	0.52 ± 0.35	0.20 ± 0.31	< 0.05	0.05 ± 0.02	0.02 ± 0.02	< 0.01
Dough, puff pastry	0.05 ± 0.10	0.00 ± 0.00	0.15	0.00 ± 0.00	0.01 ± 0.01	< 0.01	0.50 ± 0.48	0.15 ± 0.20	0.10	0.04 ± 0.04	0.01 ± 0.01	< 0.01

Notes. Values are means ± standard deviation, expressed by g/100 g edible portion of food. GC: gluten containing; GF: gluten free; *p*: non-paired significance

These rates were obtained for breads sold in Spanish supermarkets, and so they may differ from those suggested by other authors for GF breads. In Australia, Biesiekierski et al. [20] using HPLC and a different enzyme kit from the current study, determined traces of lactose in white wheat breads (taken as a reference for GC bread), while this compound was not detected in GF breads. They also obtained total fructan content of 0.19 g/100 g EPF for GF bread, which increased to 0.68 g/100 g EPF for the GC bread. Another study that collected values for GF breads was conducted by Chumpitazi et al. in the United States of America [7]. In their analyses performed entirely with commercial kits (including the same fructan kit as in the Australian approach), GF bread showed higher excess fructose values (0.25 g/100 g EPF) than ours (our values were 0.03 g/100 g EPF). Nonetheless, the levels of fructan and raffinose were closer to the average obtained in our research (0.06 and 0.03 g/100 g, EPF respectively).

The flours used as well as other ingredients (including additives), bread manufacturing itself, or the measurement techniques used for the quantification of FODMAPs may be partly responsible for the observed discrepancies. For instance, the use of ingredients such as high fructose corn syrup (HFCS) or sorbitol can result in high FODMAP breads. The second largest user of HFCS is the bread sector, but it is also widely used in juices, soft drinks, breakfast cereals and dairy desserts [21]. The use of HFCS has increased in recent years due to its affordability, sweetness and ability to enhance flavour and shelf life. The incorporation of sorbitol into bread formulations has been shown to increase baking absorption, bread weight, reduce specific volume and improve acceptability and shelf life [22].The research carried out by Ziegler et al. [23]. on German GC flours and breads showed that there was variability in wholemeal flours of different varieties of bread wheat (spelt, durum emmer and einkorn) with regard to the amount of glucose, raffinose and total fructans. Similarly, these authors also emphasised that the location of the cultures generated variability in their analysis, and that fermentation in breadmaking modified the quantities of fructans and excess fructose and raffinose. Among the studies that have measured the FODMAP content of foods, the one conducted by Ispiryan et al. [24] in Ireland is noteworthy. On the one hand, these researchers emphasised the possible altered estimation that can be obtained in the case of fructans through the use of different kits, specifically by not inserting an alpha-galactosidase step, or with high background noise in low fructan samples. On the other hand, using the same kit and procedure as used in the present investigation, the authors analysed GF foods including breads. For a GF white loaf, the total fructan, raffinose, stachyose and lactose were not detected, and only the sum of polyols (xylitol, sorbitol and mannitol) obtained a total amount of 0.03 g /100 g EPF. The GC equivalent bread did obtain quantifiable results for excess fructose and total fructan (0.19 and 0.14 g/100 g EPF, respectively) [24].

Not only did GF against GC breads show variations in FODMAP content in the present research, but differences were also found in other cereal-based foods frequently consumed by people following a GF diet. Significant changes were reported for sorbitol, total fructan or raffinose in the analysed groups of pasta, bakery or dough and puff pastry (Table 1). In almost all samples, the GC foods contain a higher amount of the corresponding FODMAP than their counterpart. Other authors have also previously described this fact for biscuit dough and breakfast cereals [10, 20, 24]. However, this is the first study to make a direct and systematic comparison between the choice of GF and GC in cereal-based products from the same supermarket shelf. For this purpose, like-by like unpaired statistical analysis of the samples was carried out. Although the foodstuffs were not the same in terms of composition, the availability when choosing GF or non-GF was the same, and for this reason some researchers have recommended this type of analysis when assessing the composition between GF and GC foods [25].

Apart from the studies mentioned above, no comprehensive research has been conducted on GF cereal-based foods. Findings of the present work indicate that the FODMAP amounts obtained vary from those of other European countries. Accordingly, it is relevant to describe the high values of fructans found in GF biscuits in Spain (1.82 g/100 g EPF), while in the case of the GF biscuits analysed in Ireland, these were not detectable [24]. Therefore, the need for country-specific FODMAP tables is confirmed by these data [7].

Regarding the interpretation of results obtained for low-FODMAP foods, we should be aware that they have two clear applications: one related to Food science and technology and the other to nutrition and dietetics. Firstly, the results confirm the hypothesis, that GF foods tend to have low FODMAP content [18, 26]. Bioprocessing techniques such as fermentation and germination have been reported to reduce levels of galacto-oligosaccharides, fructans and lactose [27]. In fact, the activation of endogenous seed enzymes and the use of yeasts and lactic acid bacteria among others; are strategies to produce low-FODMAP products [28]. Unfortunately, it was not possible to gain access to the manufacturing processes of the commercial foods analysed. For this reason, we decided to investigate their ingredient lists. Specific analyses of ingredients used in the manufacture of cereal-based foods have shown that GF ingredients contribute significantly to this, as they are mostly low in FODMAP [24]. With regard to the ingredients used in the preparation of measured GF samples, we observed no significant differences in the foods classified as low- or high-FODMAP (*p* = 0.99). Nevertheless, the correspondence analysis (supplementary Figure 1) revealed that the ingredients belonging to the subgroups of legumes, other proteins and humectants identified in the GF products were associated with being high-FODMAP. Specifically, lentil flour, guar seeds and pea proteins were found to contain excess oligosaccharides, as previously identified by other authors [24, 29]. In the case of one sample, the ingredient listed as a humectant on the labelling was found to have sorbitol excess, which suggests that it should have been listed as sorbitol.

Beyond the FODMAP content of foods, it is necessary to consider the application of these results to the diet and, in particular, to the low-FODMAP diet. The use of the low-FODMAP diet should be undertaken by dieticians [30], who for several years have had international cut-off points for the consideration of foods as low-FODMAP. Even though, as detailed in the comprehensive and well-developed review on the scientific and market perspective of FODMAP foods [28], only 19% of the products certified as low-FODMAP are marketed in Europe, and the vast majority of them do not have a certification logo [28]. The authors addressed the reasons why foods are low in FODMAP, indicating that more than three-quarters of low FODMAP cereal-based foods are also GF. Therefore, we aimed to rephrase the question to determine whether the proportion of low FODMAP cases differed between GF and GC equivalents. Our data, which categorised low FODMAP foods according to standardised serving weights in Spain, indicates a similar rate of being classified as high FODMAP in GF (5/25) and GC (6/25) products. Among the foods studied, it is clear that the prevalence of low FODMAP foods in GF foods is higher higher than that of high FODMAP foods.

Nevertheless, in the present study we use other approaches that reflect other possible dietary categories: maximum daily intake and individually wrapped size of products. It is important to note that the total FODMAP intake that causes symptoms can come from a single meal [31]. On this basis, we wanted to hypothesise what would happen if the maximum daily intake of studied-foods by the Spanish population occurred in a single meal or sitting. With the exception of baguette-type bread, the scenario would be better as the intake amount of the rest foodstuffs when considering the maximun daily intake is lower than the when considering the standardized serving (Table S3). However, the distribution of foods classified as high in FODMAP in GF (4/25) and CG (7/25) products basing on this category remains similar to that of standardised servings.

In recent years, food companies have significantly increased portion sizes, and there is consistent evidence showing that individuals tend to consume larger amounts when presented with bigger portions compared to smaller ones [32]. Therefore, we hypothesise that individually wrapped size of products could influence food/ FODMAP intake per meal. The present research found that the grams of products individually wrapped generally exceeded the maximum daily intake for the Spanish population, but were similar to the reference -serving size (Table S3) . It is worth noting that for pizza dough, puff pastry, and dough, there was no individually wrapped size, and thus, the total amount of the small pack size was considered. This assumption may introduce bias as it does not guarantee the intake of a single person. However, this type of caloric food is often consumed at social events, where more food tends to be eaten [33]. This is particularly relevant for celiac people who may be reluctant to share food with others, as a recent study by our group showed [34].

The packaging/wrapping of GF foods acts as a barrier to avoid cross-contact with gluten, one of the main concerns of the coeliac community [34]. This means that in the case of GF foods, the scenario of assessing the ratio of high FODMAPs on the basis of individually wrapped size becomes noteworthy. This potential scenario describes that GF foods have a tendency (*p* = 0.06) to have a lower possibility of being high-FODMAP (4/25) compared to GC (10/25), as shown in supplementary Table S3. Accordingly, individually wrapped GF foods can help consumers to consume less high-FODMAP foods. However, this claim cannot be applied to all GF foods and therefore cannot be generalised. Although in the case of Spain, GF foods may be an option for FODMAP-sensitive people, our results suggest that regular and specific analyses for FODMAP quantification of GF foods sold in Spain should be carried out which would help to develop the needed low-FODMAP labelling.

## Conclusion

We have determined that when presented with the option of buying cereal-based GF or equivalent GC foods, the former generally, but no in all cases, displays lower FODMAP scores. Therefore, our findings provide essential information for FODMAP content databases of GF products in Spain.

### Electronic Supplementary Material

Below is the link to the electronic supplementary material. 

Supplementary Material 1

Supplementary Material 2

Supplementary Material 3

Supplementary Material 4

Supplementary Material 5

## Data Availability

No datasets were generated or analysed during the current study.
