# Supplementary material for: FODMAP Content Like-by-like Comparison in Spanish Gluten-free and Gluten-containing Cereal-based Products
Source: Plant Foods Hum Nutr. 2024 Apr 20;79(2):545–50. doi: 10.1007/s11130-024-01177-8 (PMC11178640; doi:10.1007/s11130-024-01177-8)
Supplement: Supplementary file 1 — Supplementary Material 1 [file 11130_2024_1177_MOESM1_ESM.docx]

FODMAP content like-by-like comparison in Spanish gluten-free and gluten-containing cereal-based products

Silvia Matias ^1^, Gesala Perez-Junkera^1^, Olaia Martínez ^1,2,3^, Jonatan Miranda^1,2,3*^, Idoia Larretxi ^1,2,3,4^, Lidia Peña^2^, María Ángeles Bustamante^2.3^, Itziar Churruca ^1,2,3^, Edurne Simón ^1,2,3^

^1^ GLUTEN3S Research Group, Department of Pharmacy and Food Science, University of the Basque Country (UPV/EHU), 01006 Vitoria-Gasteiz, Spain;

^2^ Gluten Analysis Laboratory of the University of the Basque Country (UPV/EHU), Department of Pharmacy and Food Science, University of the Basque Country (UPV/EHU), 01006 Vitoria-Gasteiz, Spain

^3^ Nutrition and Food Safety Research Group, Bioaraba Health Research Institute, 01006 Vitoria-Gasteiz, Spain

^4^ Centro Integral de Atención a Mayores San Prudencio, Ayuntamiento de Vitoria-Gasteiz, 01006 Vitoria-Gasteiz, Spain

2. Material and Methods

2.1. Food samples

Twenty-five GF labelled cereal-based products most consumed by coeliac patients in Spain were selected for the research. Information about food consumption was obtained from a food frequency questionnaire and a 24-h recall survey, which was repeated on three different days –one festive and two working days- with fifty-eight coeliacs (twenty-seven adults and thirty-one children and teenagers). All subjects gave their informed consent for inclusion before they participated in the study, the protocol of which was approved by the Ethical Committee of Clinical Research of Basque Government (PI2016069). Considering the brands detailed in surveys, samples labelled as GF were purchased from the three most popular supermarkets chains in Vitoria-Gasteiz (Spain). For each GF foodstuff, an equivalent product containing gluten was also sampled from the same supermarket where the GF product was collected. Where possible, the same brand was selected for GC products. As a result, fifty foodstuffs were sampled: six breakfast cereals, four pastas, twelve breads, six biscuits, twelve bakery products, and ten samples classified as dough and puff pastry (for more information see supplementary Table 1). Samples were stored according to the manufacturer's specifications until analysis.

2.2. FODMAP quantification

Prior to the quantification all samples were freeze-dried and ground to a fine powder. The extraction of the carbohydrates was based on the method described by Ziegler et al [1] In order to stop excessive starch hydrolysis during the extraction process and to inactivate amylase activity, an aliquot of 0.4 g lyophilized powder was thoroughly mixed with 1 mL methanol. The mixture was then homogenised at room temperature for 2 × 15 s using a probe sonicator after 20 mL of water was added. The liquid phase was separated after 5 minutes of centrifugation at 1520 × g and 20 °, and the solid residue was extracted again using 20 mL of water. After the mixed extracts were prepared to a 200 mL volume, they were filtered into an HPLC vials using a 0.45 µm polyamide syringe filter.

Quantification of lactose, fructose, glucose, sorbitol, mannitol, raffinose, and stachyose was carried out in duplicate, from the same powder, by a high-performance anion-exchange chromatographic system coupled with pulsed amperometric detection (HPAEC-PAD) following a validated method reported [(2)]. A Dionex ICS-5000+ Ion Chromatograph (Thermo Scientific, USA) was briefly used for the analysis, with two different columns, PA210 for the determination of glucose, fructose, lactose, raffinose and stachyose, and PA10 for determination of sorbitol and mannitol. In PA210, the analytical column Dionex CarboPac PA210-4 μm; 2 x 150 mm (Thermo Scientific, USA) was protected with a Dionex CarboPac PA210 G-4 μm; 2 x 30 mm guard column (Thermo Scientific, USA). Separation was performed in isocratic conditions with KOH 16 mM eluent, produced by Dionex EGC 500 KOH Eluent Generator Cartridge with Dionex CR-ATC 500 continuously regenerated by Anion Trap column (Thermo Scientific, USA). Flow rate was 0.2 mL/min and injection volume was 2.5 μL. For the measurement of mannitol and sorbitol, an analytical column of CarboPAC PA10, 4x250 mm (Thermo Scientific, USA) was used, with a guard column of CarboPAC PA10 Guard 4x50 mm (Thermo Scientific, USA). KOH 18 mM was used as the isocratic mobile phase with a flow rate of 1 mL/min and an injection volume of 10 μL. In both cases, an electrochemical detector with a gold working electrode and a pH reference electrode, was used. Finally, the chromatogram time was 35 min for PA210, against 40 min for PA10.

External calibration was performed with multi-elemental standards at concentrations between 0.2 and 30 mg/kg for glucose, fructose, lactose, raffinose and stachyose standards, and between 0.5 and 20 mg/kg for sorbitol and mannitol standards (Merck KGaA, Germany). The limit of quantification (LOQ) was established at 0.2 mg/kg for glucose, fructose, lactose, raffinose and stachyose, and at 0.5 mg/kg for sorbitol and mannitol.

As the chromatographic analyses were performed on freeze-dried samples, the moisture content percentage of the samples was calculated according to AOAC official method (AOAC 945.15-1945). Results were expressed per 100g edible portion of food (EPF).

Total fructan analysis was carried out using the K-FRUC kit (Megazyme, Ireland) following the manufacturer's instructions. All foodstuffs were measured in duplicate and each replicate was processed independently. As indicated in the protocol, to avoid overestimation of fructans by galactosyl-sucrose oligosaccharides, an incubation step with α-galactosidase from *A. niger* (Megazyme, Ireland) was included in the procedure.

2.3.Dietary assessment of FODMAP

After assessing the content of each FODMAP analysed per 100 g of EPF, the food was categorised as high or low in FODMAPs based on the cut-off points established by Varney *et al.* [3]. For this purpose, the FODMAP contents were expressed per food serving size (grams). The Healthy Eating Guide by the Spanish Society of Community Nutrition (SENC) was used as a reference for serving sizes [4]. Similarly, FODMAP content data were also expressed as the maximun quantity consumed per day and reported by the Spanish adult population [5] and the individually wrapped size of each food. In both cases, the cut-off points stated by Varney *et al.* [3] for typical serving size were used for the categorisation of foods as high-FODMAP. The cut-off values for each FODMAP carbohydrate (per serving of food per meal) are as follows: < 0.3g per serving for oligosaccharides (fructan and α-galacto-oligosaccharides), < 0.15g per serving for fructose in excess to glucose, < 0.2g per serving for sorbitol or mannitol, < 0.4g per serving for total polyols, and < 1.0g per serving for lactose. Since the reliability of the cut-off points was confirmed in different dietary studies in a single sitting or meal, they were assumed to be adequate for the purpose of the present study. Some of the products, such as pizza dough, puff pastry, and dough, were not individually wrapped. As a result, the smallest available size was selected. For instance, it was assumed that a small pizza would be suitable for one person. In the case of pasta and breakfast cereals, the recommended serving size was taken as reference.

2.4.Food ingredient analysis

A database was created with the ingredients of each foodstuff. The ingredients were categorized into the following groups: cereals, legumes, non-grass plants, tubers, egg protein, milk protein, other protein, water, sugars, fats and oils, fibres, salt/vitamins/minerals and fruits/nuts. An additional group was created for additives. This group was divided into eight subgroups according to the functional classes from the Codex General Standard for Food Additives (GSFA) Online Database: thickeners, foaming agents, emulsifiers, acidity regulators/antioxidants, humectants, flavour enhancers, sweeteners and other additives. With all this information, a correspondence analysis was performed between the samples with high FODMAP classification and their ingredients.

2.5. Data treatment and statistical analysis

The results were given as the arithmetic mean ± standard deviation. Taking into account that FODMAP content variables followed a skewed distribution, non-paired Mann-Whitney U tests was used to compare GF and CG products. Duplicates were considered as different samples for the application of the test. The χ2 test was performed to determine differences in frequencies of high-FODMAP categorized variable between groups. IBM SPSS Statistics software version 28.0 (New York, USA) was used for those statistical analysis. The correspondence analysis between food ingredients and the classification of GF products as high- or low-FODMAP was performed with the Real Statistics Resource Pack software (Release 7.6) in Excel (Charles Zaiontz. [www.real-statistics.com](http://www.real-statistics.com)). Significance of correspondence was checked by applying the χ2 test and maximum likelihood statistic. In all cases, a significance level was set at *p* ≤ 0.05.

3. References

1. Ziegler JU, Steiner D, Longin CFH et al. (2016) Wheat and the irritable bowel syndrome – FODMAP levels of modern and ancient species and their retention during bread making. Journal of Functional Foods 25, 257-266 doi: 10.1016/j.jff.2016.05.019

2. Ispiryan L, Heitmann M, Hoehnel A et al. (2019) Optimization and Validation of an HPAEC-PAD Method for the Quantification of FODMAPs in Cereals and Cereal-Based Products. J Agric Food Chem 67, 4384-4392 doi: 10.1021/acs.jafc.9b00382

3. Varney J, Barrett J, Scarlata K et al (2017) FODMAPs: food composition, defining cutoff values and international application. J Gastroenterol Hepatol 32 Suppl 1, 53-61 doi:10.1111/jgh.13698

4. Aranceta-Bartrina J, Partearroyo T, López-Sobaler AM et al (2019) Updating the Food-Based Dietary Guidelines for the Spanish Population: The Spanish Society of Community Nutrition (SENC) Proposal. Nutrients 11, 5 doi: 10.3390/nu11112675

5. European Food Safety Authority (2022) The EFSA comprehensive european food consumption database. European Union Web. http:// https://www.efsa.europa.eu/en/data-report/food-consumption-data. Accessed 12 December 2023
