# Supplementary material for: FODMAP Content Like-by-like Comparison in Spanish Gluten-free and Gluten-containing Cereal-based Products
Source: Plant Foods Hum Nutr. 2024 Apr 20;79(2):545–50. doi: 10.1007/s11130-024-01177-8 (PMC11178640; doi:10.1007/s11130-024-01177-8)
Supplement: Supplementary file 2 — Supplementary Material 2 [file 11130_2024_1177_MOESM2_ESM.docx]

Supplementary table 1. Analysed FODMAP content by each sample.

|  | g/100g edible portion of food | | | | | | | | |
| --- | --- | --- | --- | --- | --- | --- | --- | --- | --- |
| **Food** | **Lactose** | **Fructose** | **Glucose** | **Sorbitol** | **Mannitol** | **Raffinose** | **Stachyose** |  | **Fructans** |
| Biscuit GC 1 | 0.01 ± 0.00 | ND | 0.64 ± 0.00 | ND | ND | 0.26 ± 0.01 | ND | 1.08 ± 0.00 | |
| Biscuit GC 2 | 0.01 ± 0.00 | ND | 0.33 ± 0.06 | ND | UL | 0.14 ± 0.02 | 0.01 ± 0.00 | 1.11 ± 0.01 | |
| Biscuit GC 3 | 0.70 ± 0.01 | ND | 1.27 ± 0.18 | ND | ND | 0.21 ± 0.00 | ND | 0.92 ± 0.01 | |
| Biscuit GF 1 | UL | ND | 1.51 ± 0.31 | 0.02 ± 0.00 | ND | 0.31 ± 0.00 | ND | 0.17 ± 0.00 | |
| Biscuit GF 2 | 0.01 ± 0.00 | ND | 0.41 ± 0.05 | ND | UL | 0.13 ± 0.01 | 0.01 ± 0.00 | 5.07 ± 0.00 | |
| Biscuit GF 3 | 0.68 ± 0.00 | ND | 0.36 ± 0.03 | ND | ND | 0.01 ± 0.00 | ND | 0.23 ± 0.00 | |
| Bread GC 1 | ND | 0.23 ± 0.00 | 0.08 ± 0.00 | UL | ND | 0.09 ± 0.00 | ND | 0.33 ± 0.01 | |
| Bread GC 2 | ND | 0.17 ± 0.01 | 0.06 ± 0.00 | UL | UL | 0.10 ± 0.03 | ND | 0.23 ± 0.00 | |
| Bread GC 3 | ND | 0.21 ± 0.00 | 0.06 ± 0.00 | UL | ND | 0.06 ± 0.00 | ND | 0.14 ± 0.00 | |
| Bread GC 4 | ND | 0.43 ± 0.01 | 0.16 ± 0.01 | UL | ND | 0.12 ± 0.05 | ND | 0.38 ± 0.01 | |
| Bread GF 1 | 0.01 ± 0.00 | 0.05 ± 0.00 | 2.81 ± 0.34 | UL | ND | ND | ND | ND | |
| Bread GF 2 | 0.00 ± 0.00 | 0.53 ± 0.07 | 0.90 ± 0.09 | ND | ND | ND | ND | ND | |
| Bread GF 3 | ND | 1.40 ± 0.00 | 1.41 ± 0.02 | UL | UL | 0.08 ± 0.00 | ND | ND | |
| Bread GF 4 | 0.01 ± 0.00 | 0.27 ± 0.02 | 0.18 ± 0.01 | ND | UL | ND | ND | ND | |
| Breakfast cereals GC 1 | UL | 1.67 ± 0.03 | 1.90 ± 0.08 | UL | 0.04 ± 0.00 | 0.05 ± 0.00 | ND | 0.14 ± 0.03 | |
| Breakfast cereals GC 2 | 0.02 ± 0.00 | 6.87 ± 0.93 | 6.59 ± 0.95 | 0.16 ± 0.01 | 0.01 ± 0.00 | 0.13 ± 0.01 | 0.04 ± 0.01 | 0.43 ± 0.01 | |
| Breakfast cereals GF 1 | UL | 0.42 ± 0.02 | 0.48 ± 0.02 | 0.04 ± 0.00 | ND | 0.01 ± 0.00 | ND | ND | |
| Breakfast cereals GF 2 | 0.01 ± 0.00 | 13.5 ± 0.45 | 13.9 ± 0.48 | 0.16 ± 0.00 | 0.02 ± 0.00 | 0.15 ± 0.01 | 0.67 ± 0.01 | 0.47 ± 0.03 | |
| Bun GC 1 | ND | 0.35 ± 0.03 | 0.11 ± 0.01 | UL | ND | 0.06 ± 0.01 | ND | 0.43 ± 0.02 | |
| Bun GC 2 | ND | 1.02 ± 0.09 | 1.16 ± 0.10 | 0.51 ± 0.02 | UL | 0.04 ± 0.00 | ND | 1.17 ± 0.00 | |
| Bun GF 1 | 0.01 ± 0.00 | 0.15 ± 0.00 | 3.52 ± 0.34 | ND | ND | ND | ND | ND | |
| Bun GF 2 | ND | 4.83 ± 0.48 | 4.30 ± 0.40 | 0.65 ± 0.01 | ND | 0.06 ± 0.00 | ND | 0.00 ± | |
| Cake GC 1 | ND | ND | 0.65 ± 0.16 | 1.66 ± 0.07 | 0.07 ± 0.00 | 0.10 ± 0.00 | ND | 0.57 ± 0.04 | |
| Cake GF 1 | ND | ND | 0.51 ± 0.01 | 0.24 ± 0.00 | ND | 0.03 ± 0.00 | ND | 0.74 ± 0.01 | |
| Cereal bar GC 1 | UL | 0.77 ±0.08 | 5.30 ± 0.14 | 1.16 ± 0.13 | UL | 0.15 ± 0.01 | 0.05 ± 0.00 | 0.50 ± 0.01 | |
| Cereal bar GF 1 | 0.05 ± 0.01 | 5.71 ±0.49 | 10.2 ± 0.70 | 3.57 ± 1.54 | UL | 0.17 ± 0.01 | 0.10 ± 0.02 | 0.41 ± 0.04 | |
| Croissant GC 1 | ND | 2.25 ±0.24 | 2.02 ± 0.21 | 0.00 ± 0.00 | ND | 0.06 ± 0.01 | ND | 0.77 ± 0.00 | |
| Croissant GF 1 | ND | 0.75 ±0.14 | 4.53 ± 0.61 | 0.01 ± 0.00 | ND | 0.01 ± 0.00 | UL | 0.29 ± 0.01 | |
| Hamburger bun GC 1 | ND | 2.00 ±0.06 | 1.22 ± 0.05 | UL | ND | 0.04 ± 0.00 | ND | 0.23 ±0.01 | |
| Hamburger bun GF 1 | 0.01 ± 0.00 | 0.40 ± 0.15 | 0.66 ± 0.23 | ND | UL | ND | ND | ND | |
| Muffin GC 1 | ND | ND | 0.04 ±0.00 | 0.94 ± 0.04 | UL | 0.04 ± 0.00 | ND | 0.61 ± 0.04 | |
| Muffin GF 1 | ND | ND | 0.05 ± 0.00 | ND | ND | 0.01 ± 0.00 | ND | 0.49 ± 0.00 | |
| Pasta GC 1 | ND | 0.06 ± 0.01 | 0.11 ± 0.00 | ND | ND | 0.12 ± 0.00 | ND | 0.75 ± 0.01 | |
| Pasta GF 1 | UL | 0.07 ± 0.01 | 0.12 ± 0.00 | 0.03 ± 0.01 | ND | 0.03 ± 0.00 | ND | ND | |
| Pasta GC 2 | UL | 0.00 ± 0.00 | 0.18 ± 0.01 | 0.01 ± 0.00 | ND | 0.04 ± 0.00 | ND | 0.66 ± 0.02 | |
| Pasta GF 2 | 0.01 ± 0.00 | ND | 0.43 ± 0.05 | 0.01 ± 0.00 | ND | 0.05 ± 0.00 | ND | 0.12 ± 0.01 | |
| Pastry based on laminated dough GC 1 | ND | ND | 1.48 ± 0.22 | ND | 0.04 ± 0.00 | 0.11 ± 0.00 | 0.02 ± 0.00 | 1.25 ± 0.03 | |
| Pastry based on laminated dough GF 1 | ND | 2.98 ± 0.50 | 6.01 ± 1.10 | ND | ND | 0.03 ± 0.00 | 0.05 ± 0.00 | 0.46 ± 0.01 | |
| Pie dough GC 1 | ND | 0.27 ± 0.02 | 0.31 ± 0.01 | ND | ND | 0.01 ± 0.00 | ND | 0.28 ± 0.01 | |
| Pie dough GF 1 | ND | 0.09 ± 0.00 | 1.19 ± 0.63 | UL | ND | UL | ND | ND | |
| Pizza dough GC 1 | UL | 0.37 ± 0.00 | 0.85 ± 0.19 | ND | ND | 0.03 ± 0.00 | ND | ND | |
| Pizza dough GF 1 | ND | 0.02 ± 0.00 | 1.38 ± 0.01 | ND | ND | ND | ND | ND | |
| Puff Pastry GC 1 | 0.03 ± 0.01 | 0.19 ± 0.04 | 0.30 ± 0.07 | ND | ND | 0.01 ± 0.00 | ND | 0.21 ± 0.01 | |
| Puff pastry GF 1 | ND | 0.02 ± 0.00 | 2.07 ± 0.04 | UL | UL | ND | ND | ND | |
| Sandwich bread GC 1 | ND | 0.37 ± 0.01 | 2.28 ± 0.06 | UL | ND | 0.03 ± 0.00 | ND | 0.13 ± 0.00 | |
| Sandwich bread GF 2 | 0.01 ± 0.00 | 0.03 ± 0.00 | 1.58 ± 0.27 | UL | ND | ND | ND | ND | |
| Toasted bread GC 1 | 0.01 ± 0.00 | 0.48 ± 0.02 | 0.18 ± 0.01 | UL | ND | 0.24 ± 0.03 | ND | 0.44 ± 0.06 | |
| Toasted bread GC 2 | ND | 1.14 ± 0.03 | 0.38 ± 0.00 | UL | ND | 0.24 ± 0.02 | ND | 0.33 ± 0.01 | |
| Toasted bread GF 1 | UL | 0.10 ± 0.02 | 0.91 ± 0.20 | ND | ND | 0.01 ± 0.00 | ND | ND | |
| Toasted bread GF 2 | ND | 2.93 ± 0.33 | 2.80 ± 0.34 | UL | ND | 0.07 ± 0.00 | ND | 0.54 ± 0.03 | |

Notes. ND: not detectable; UD; under limit.
