# Supplementary material for: FODMAP Content Like-by-like Comparison in Spanish Gluten-free and Gluten-containing Cereal-based Products
Source: Plant Foods Hum Nutr. 2024 Apr 20;79(2):545–50. doi: 10.1007/s11130-024-01177-8 (PMC11178640; doi:10.1007/s11130-024-01177-8)
Supplement: Supplementary file 3 — Supplementary Material 3 [file 11130_2024_1177_MOESM3_ESM.docx]

Supplementary table 2. Analytical lactose, mannitol and stachyose content in gluten-free foodstuffs divided by groups, compared to gluten-containing products.

| **Food group** | **Lactose** | | | **Mannitol** | | | **Stachyose** | | |
| --- | --- | --- | --- | --- | --- | --- | --- | --- | --- |
|  | GC | GF | *p* | GC | GF | *p* | GC | GF | *p* |
| Breakfast cereals | 0.01 0.01 | 0.02±0.02 | 0.11 | 0.02 ± 0.02 | 0.01 ± 0.01 | 0.33 | 0.03±0.02 | 0.26±0.33 | 0.09 |
| Pasta | 0.00±0.00 | 0.01±0.00 | <0.05 | 0.00 ±0.00 | 0.00 ± 0.00 | 1.00 | 0.00±0.00 | 0.00±0.00 | 1.00 |
| Bread | 0.00±0.00 | 0.00±0.00 | <0.05 | 0.00 ±0.00 | 0.00 ±0.00 | 0.54 | 0.00±0.00 | 0.00±0.00 | 1.00 |
| Biscuits | 0.24±0.36 | 0.23±0.35 | 0.33 | 0.00 ± 0.00 | 0.00 ± 0.01 | 0.70 | 0.00±0.00 | 0.00±0.00 | 0.85 |
| Bakery | 0.00±0.00 | 0.00±0.00 | <0.01 | 0.02 ±0.03 | 0.00 ±0.00 | <0.05 | 0.00±0.00 | 0.00±0.00 | 1.00 |
| Dough, puff pastry | 0.01±0.01 | 0.00±0.00 | <0.05 | 0.01 ± 0.02 | 0.00 ± 0.00 | 0.83 | 0.00±0.01 | 0.01±0.02 | 0.35 |

Notes. Values are means ± standard deviation, expressed by g/100g edible portion of food. GC: gluten containing; GF: gluten free; *p*: non-paired significance
