# Supplementary material for: FODMAP Content Like-by-like Comparison in Spanish Gluten-free and Gluten-containing Cereal-based Products
Source: Plant Foods Hum Nutr. 2024 Apr 20;79(2):545–50. doi: 10.1007/s11130-024-01177-8 (PMC11178640; doi:10.1007/s11130-024-01177-8)
Supplement: Supplementary file 4 — Supplementary Material 4 [file 11130_2024_1177_MOESM4_ESM.docx]

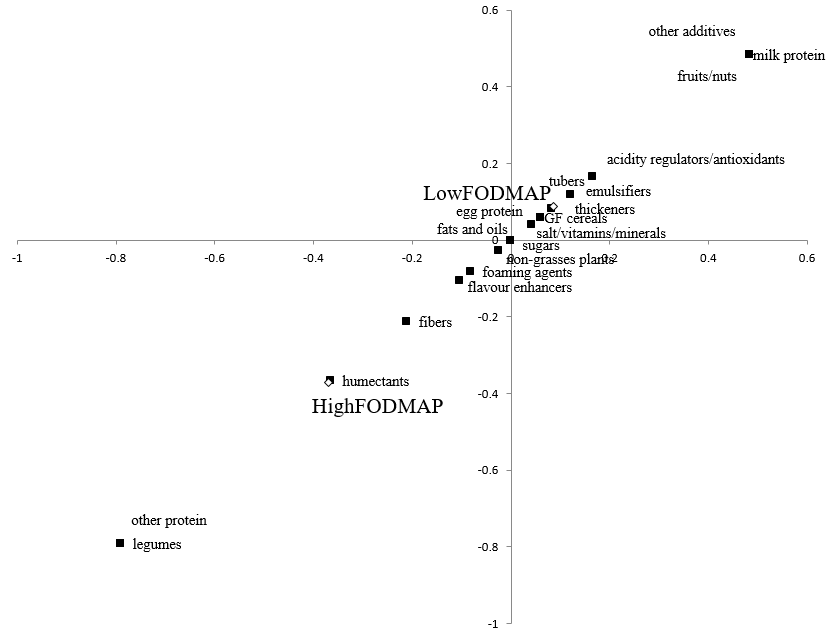


Supplementary figure 1. Correspondence analysis between the samples with high FODMAP classification and their ingredients.
