# Supplementary material for: FODMAP Content Like-by-like Comparison in Spanish Gluten-free and Gluten-containing Cereal-based Products
Source: Plant Foods Hum Nutr. 2024 Apr 20;79(2):545–50. doi: 10.1007/s11130-024-01177-8 (PMC11178640; doi:10.1007/s11130-024-01177-8)
Supplement: Supplementary file 5 — Supplementary Material 5 [file 11130_2024_1177_MOESM5_ESM.docx]

Supplementary table 3. Number of samples classified as high-FODMAP (food expressed by serving size, maximum food intake per day or individually wrapped size).

| **Food group** | **n** | **Serving Size (g)** | **High FODMAP** | | **p** | **Max food intake (g)** | **High FODMAP** | | **p** | **Individually Wrapped Size (g)** | **High FODMAP** | | **p** |
| --- | --- | --- | --- | --- | --- | --- | --- | --- | --- | --- | --- | --- | --- |
|  |  |  | GC | GF |  |  | GC | GF |  |  | GC | GF |  |
| Biscuit | 6 | 50 | 1 | 1 |  | 31 | 1 | 1 |  | 48 | 1 | 1 |  |
| Breakfast cereals | 4 | 40 | 1 | 0 |  | 28 | 1 | 0 |  | 40 | 1 | 0 |  |
| Cake | 2 | 80 | 1 | 1 |  | 40 | 1 | 1 |  | 31 | 1 | 0 |  |
| Cereal bar | 2 | 30 | 0 | 0 |  | 23 | 0 | 0 |  | 29 | 0 | 0 |  |
| Croissant | 2 | 50 | 0 | 0 |  | 46 | 0 | 0 |  | 32 | 0 | 0 |  |
| Dough | 2 | 30 | 0 | 0 |  | 15 | 0 | 0 |  | 120 | 1 | 0 |  |
| Hamburger bun | 6 | 60 | 0 | 1 |  | 39 | 0 | 1 |  | 81 | 0 | 1 |  |
| Heart-shaped puff pastry | 2 | 50 | 1 | 0 |  | 35 | 1 | 0 |  | 38 | 1 | 0 |  |
| Muffin | 2 | 50 | 0 | 0 |  | 21 | 0 | 0 |  | 34 | 0 | 0 |  |
| Pasta | 4 | 80 | 0 | 0 |  | 36 | 0 | 0 |  | 80 | 0 | 0 |  |
| Pizza dough | 2 | 53 | 0 | 0 |  | 52 | 0 | 0 |  | 128 | 1 | 0 |  |
| Puff pastry | 2 | 50 | 0 | 0 |  | 36 | 0 | 0 |  | 130 | 0 | 0 |  |
| Sliced bread | 2 | 60 | 1 | 0 |  | 39 | 1 | 0 |  | 30 | 1 | 0 |  |
| Toasted bread | 4 | 60 | 0 | 1 |  | 14 | 0 | 0 |  | 66 | 0 | 1 |  |
| Baguette type bread | 8 | 60 | 1 | 1 |  | 85 | 2 | 1 |  | 108 | 3 | 1 |  |
| TOTAL | 50 |  | 6 | 5 | 0.73 |  | 7 | 4 | 0.30 |  | 10 | 4 | 0.06 |

Notes. Each food was categorised as high or low in FODMAPs based on the cut-off points established by Varney *et al.* [17]. GC: gluten containing; GF: gluten free; *p*: significance
